# Cyclitis and Irido-Cyclitis

**Published:** 1908-08-15

**Authors:** 


### Ophthalmology.

### CYCLITIS AND IRIDO-CYCLITIS—III.

### TREATMENT.

The treatment of cyclitis resolves itself into remedial measures directed against the constitutional cause, and towards relief of the local condition; the latter may have to be varied during the course of an attack to meet the various symptoms as they arise, such as the intolerable neuralgic pain and glaucomatous tension.

#### General Treatment.

Constitutionally, specific disease should be dealt with vigorously by inunction of the oleate or other preparation of mercury until the patient is well under its influence; when this has been successfully brought about the effect should be kept up by occasional inunctions and by potassium iodide given three times a day. If the patient gets into a very depressed state the latter should be combined with tinct. nucis vomicæ, but at all costs it must be continued.

Where rheumatism appears to be the cause, aspirin should be given with quinine, failing this salicine, or salicylate of soda, but always with quinine. Tuberculosis should be treated with cod-liver oil, the usual anti-tubercular remedies, and at the same time with injections of tuberculin over a considerable period; these cases extend well over twelve months, are very chronic, and react but slowly to remedies. Cases of interstitial keratitis and accompanying cyclitis are best treated with syrup of the iodide of iron.

Where septic infection from the mouth and teeth is the cause of the cyclitis, all decayed teeth and stumps should first be removed, alveolar abscesses drained, etc., and a mouth-wash of chlorate of potash used. In all those cases in which obstinate constipation or auto-infection appears to be the only cause, it is well to begin with calomel and to continue with pulv. hyd. c̄ creta, gr. 1½, every other night.

Calomel should also be given in cases of sympathetic ophthalmia; mercury in some form seems to be the only remedy which exerts any beneficial action upon this particular inflammation.

#### Local Treatment.

Locally, the most important indication is dilatation of the pupil by atropine; first because it puts the iris and ciliary body at rest, distinctly lessening pain, and to some extent congestion, for atropine also acts as a direct sedative absorbed locally; secondly, because when the pupil is fully dilated the iris is drawn away from the lens capsule and runs less risk of becoming adherent to it, the posterior chamber is less liable to become filled with exudate, and seclusion and occlusion of the pupil are prevented.

Congestion of the eye is reduced by blood-letting by means of leeches applied to the temple, or by means of Heurteloup's artificial leech. It is frequently found after blood-letting that a pupil which hitherto has not dilated fully under atropine, rapidly relaxes and is easily kept dilated by an occasional instillation of that drug. The relief of the congestion generally means the relief of the pain, and if the pain returns, leeches should be again applied. In mild cases a blister, one inch square, applied to the temple will act as well as leeches.

To relieve the pain, hot fomentations continuously applied will at times be all that is necessary; cold applications rarely give the slightest relief and occasionally cause an exacerbation of the neuralgia. The greatest comfort and the most beneficial action is brought about by the application of dry heat for several hours at a time. The simplest method of applying this is by a small metal box filled with some slowly-smouldering material after the style of the "instra" muff warmers, a pad of fair thickness is first placed over the eye, then the box with the material already smouldering inside it, and this is covered by a light pad, the whole kept in position by a bandage. Where the electric current is available, continuous heat may be obtained by a small asbestos pad containing a zig-zag wire, each end of which is connected to the current; by means of a resistance box the current and thus the heat are regulated at the same time. The zig-zag wire is never allowed to reach a red heat or anything approaching it. but just sufficiently hot to warm the whole pad and eye to a temperature comfortable for the patient, and kept at this for a couple of hours at a time. A very convenient pad and regulating apparatus of this kind has been devised by Maddox solely for eye cases.

Massage of the supra-orbital region with an ointment of gr. ¼ morphine to the drachm of vaseline will, at times, ease the pain. If the tension of the eye goes up, atropine should be still used, but less frequently until the pupil is fully dilated, and if the tension reaches +1 or over, paracentesis of the anterior chamber should be performed.

During an attack of cyclitis the inflamed eye should be kept bandaged, and the other as much at rest as possible by dark protectors; no reading is to be permitted under any consideration. No alcohol should be taken and as little meat as possible. The bowels also may with advantage be kept freely acting, without any severe purgation.

Cyclitis is very apt to relapse after the first attack has quieted down, and after an attack any discomfort and redness of the eye which may appear should be at once treated by atropine, unless, of course, the tension is high. Secondary glaucoma occurring after cyclitis, due to the adhesion of the iris to the lens capsule and obliteration of the posterior chamber, should be treated by iridectomy six to eight weeks after the attack of cyclitis, and only then if all inflammatory symptoms have subsided.

